# Data-Driven Multidimensional Performance Profiling in U10 Tennis Players

**DOI:** 10.3390/sports14090382

**Published:** 2026-09-01

**Authors:** Rita Géczi, Csaba Ökrös, Károly Dobos, Katalin Orbán-Sebestyén, Nóra Papp, László Tóth

**Affiliations:** 1 School of Doctoral Studies, Hungarian University of Sports Science, 1123 Budapest, Hungary; 2 Department of Sport Games, Hungarian University of Sports Science, 1123 Budapest, Hungary; 3 Eötvös Loránd University Faculty of Education and Psychology, Institute of Health Development and Sport Sciences, 1075 Budapest, Hungary; 4 Department of Psychology and Sports Psychology, Hungarian University of Sports Science, 1123 Budapest, Hungary; 5 Teacher Training Institute, Hungarian University of Sports Science, 1123 Budapest, Hungary; 6 Institute of Health Promotion and Sport Sciences, Faculty of Education and Psychology, Eötvös Loránd University, 1053 Budapest, Hungary

**Keywords:** talent identification, youth tennis, cluster analysis, principal component analysis, multidimensional profiling, physical performance

## Abstract

Objective: Talent identification in youth tennis remains challenging because current assessment approaches often evaluate physical, technical, and cognitive characteristics separately rather than within an integrated multidimensional framework. The present study aimed to identify multidimensional performance profiles in U10 tennis players by integrating technical, physical-motor, and reaction-based cognitive performance characteristics within a single analytical framework. Methods: A total of 113 players participating in the Hungarian Tennis Federation’s U10 Talent Identification Festival were assessed using a multidimensional performance battery. Following data cleaning, 113 participants were included in the analyses. Principal component analysis was used to examine the dimensional structure of the assessment battery, whereas k-means clustering was performed separately on the ten standardized performance variables. The optimal number of clusters was determined using complementary validation procedures, including the elbow method, average silhouette width, and gap statistic. Differences between the two performance profiles were examined using Welch’s *t*-tests with Hedges’ *g* effect sizes, while associations of profile membership with birth quarter and sex were assessed using chi-square analyses. An age-adjusted sensitivity analysis was additionally performed to evaluate the influence of chronological age on the clustering solution. Results: A two-cluster solution was retained, comprising a higher-performing profile (n = 48) and a lower-performing profile (n = 65). The higher-performing profile demonstrated better performance across all ten technical, physical-motor, and reaction-based measures, with the largest standardized differences observed for throwing speed (Hedges’ *g* = 1.43), technique (*g* = 1.42), and 15-m sprint performance (|g| = 1.38). Chronological age differed significantly between profiles, with participants in the higher-performing profile being older (9.41 vs. 8.80 years; Welch’s t(102.94) = 4.24, *p* < 0.001; Hedges’ *g* = 0.80). However, the two-cluster solution remained highly stable after age adjustment (bootstrap Jaccard coefficients = 0.937 and 0.946), with 85.0% correspondence in cluster membership. Neither birth quarter (χ^2^(3) = 0.28, *p* = 0.964) nor sex (χ^2^(1) = 3.19, *p* = 0.074) was significantly associated with profile membership. Conclusions: Multidimensional profiling identified two distinct performance profiles in U10 tennis players, primarily reflecting a broad gradient in current technical, physical-motor, and reaction-based performance. Although chronological age contributed to profile membership, the persistence of the cluster structure after age adjustment suggests that age did not fully account for the observed performance heterogeneity. These profiles should be interpreted as exploratory descriptions of current multidimensional performance rather than fixed talent categories or predictors of future development. In practice, multidimensional assessment may help coaches identify relative strengths and weaknesses across performance domains and support individualised player development; however, profile membership should not be used as a stand-alone criterion for talent selection or deselection. Longitudinal studies are needed to establish their developmental stability and predictive validity.

## 1. Introduction

Talent identification (TID) is widely recognised as a cornerstone of long-term athlete development in tennis and in organised youth sport more broadly [1], with national federations implementing age-graded selection programmes from as early as the U8 category [2]. At each successive stage, resources, competitive opportunities, and coaching expertise are progressively concentrated on players deemed developmentally promising, rendering early identification decisions consequential not only for individual athletes but for the long-term performance of national systems [3]. This responsibility is compounded by the inherently heterogeneous nature of talent development: prospective longitudinal evidence suggests that talented players follow multiple distinct developmental trajectories rather than a single normative pathway, and that the most successful performers do not always emerge as the most advanced at the earliest point of assessment [3,4,5]. Against this background, the predictive accuracy of current TID tools remains limited. Studies applying logistic regression, discriminant analysis, and neural networks to early-age test batteries have demonstrated only medium-to-high prognostic validity, with up to 18.75% of future elite performers remaining unidentifiable at initial screening regardless of statistical method [6]. This ceiling in predictive performance points to a need for broader, multidimensional assessment frameworks—beginning with the physical performance characteristics that have attracted greatest empirical attention in the tennis TID literature.

Physical and motor performance testing has formed the empirical bedrock of youth tennis TID research, and its contribution is substantial [7,8]. Variables including linear sprint speed, explosive lower-limb power, throwing velocity, agility, and motor coordination have each demonstrated discriminative capacity across age-graded samples, with selection odds ratios ranging from 0.18 to 7.50 in U8–U13 players assessed within a structured national development programme [2]. Specific tests—notably the standing broad jump, ball throw, and endurance run—show meaningful prognostic associations with junior competitive outcomes at follow-up intervals of four years or more, with physical characteristics exhibiting relative stability between U9 and late junior age [6]. More recently, both generic and tennis-specific agility assessments have demonstrated high reliability alongside significant discriminative validity in U12 players, reinforcing the value of multidirectional speed measures within TID batteries [9]. Collectively, this evidence confirms that physical profiling captures a meaningful component of early tennis potential.

However, its explanatory scope is demonstrably incomplete. The prognostic validity of physical test batteries remains bounded at moderate levels, and recent meta-analytic synthesis has explicitly identified the U8–U10 age bracket as underrepresented in the existing evidence base [10]. Although physical capacities provide an important foundation for tennis performance, competitive success is ultimately expressed through the effective execution of technical skills during match play [8,11]. Furthermore, physical test batteries cannot capture the stroke execution, ball control, and shot quality that fundamentally define tennis performance [11]—an omission identified as an increasingly recognised limitation in youth TID, and one that motivates the need for validated technical skill assessment in early performance profiling.

Technical proficiency is widely regarded as one of the defining sport-specific performance domains in tennis [8,11], where the capacity to execute strokes with consistency, directional precision, depth control, and spin variation ultimately determines competitive outcomes. Accordingly, technical assessment should represent a central component of early talent identification programmes. Despite this centrality, technical skill has been systematically underrepresented in TID research. Where technical variables have been incorporated, assessment has typically been reduced to binary outcome measures—whether a ball lands within a designated court area or not—capturing basic consistency while providing no information about landing depth, directional accuracy, net clearance, or spin characteristics [12]. This reductionism limits the discriminative resolution of TID batteries, rendering existing tools poorly suited to distinguishing players at different stages of technical development. Recent literature has identified this as a structural constraint: Oliveira et al. [13] explicitly called for the integration of multidimensional technical variables into cluster-based profiling frameworks for young tennis players—a call that remains unanswered in the published literature. This methodological approach has been demonstrated in adjacent racket sports: Faber et al. [12] developed and validated a multidimensional technical assessment tool for youth table tennis players aged 8–12, incorporating stroke quality, footwork, and service evaluation with explicit criteria distinguishing flawed from excellent execution. To the best of current knowledge, no comparable multidimensional technical assessment instrument has been reported within the youth tennis TID literature.

If technical skill has been structurally marginalised in early talent assessment, reaction-based cognitive performance has received even less attention. Perceptual-cognitive expertise is increasingly recognised as an important contributor to sport performance, with meta-analytic evidence indicating that higher-skilled athletes outperform lower-skilled counterparts across domains such as sport-specific decision-making, anticipation, and information processing [14,15]. However, these complex, sport-specific perceptual-cognitive abilities should be distinguished from the more generic reaction-based performance assessed in the present study. The BlazePod task used here primarily captures rapid stimulus detection, response selection, and motor response under a relatively simple visual stimulus-response condition; it does not directly assess tennis-specific anticipation or decision-making. Nevertheless, rapid detection and response to visual stimuli represent relevant elementary components of information processing in tennis, a sport characterised by constrained response windows [16,17]. Evidence from youth sport further indicates that perceptual-cognitive and related information-processing abilities continue to develop throughout late childhood and adolescence and are responsive to practice [18,19]. Accordingly, the reaction-based measure included in the present battery should be interpreted as a limited reaction-based cognitive-perceptual indicator within a broader multidimensional assessment framework rather than as a comprehensive measure of perceptual-cognitive expertise.

Addressing these interrelated gaps requires an integrated, multidimensional framework—one in which the developmental characteristics of the target age group and the statistical method used to model performance heterogeneity receive explicit justification.

The U10 age category represents an important developmental period for talent identification in tennis. Most players in this age category are expected to remain pre-PHV, and the maturational acceleration that becomes increasingly influential during adolescence may therefore be less pronounced than in older age groups [20,21]. Nevertheless, meaningful inter-individual differences in chronological age, biological maturation, body size, training exposure, and developmental experience may already exist during late childhood and can influence performance. Accordingly, U10 should not be considered free from maturational or developmental confounding. Rather, it provides an opportunity to examine multidimensional performance characteristics during a period in which the influence of biological maturation may be less pronounced than during adolescence.

A further consideration concerns the validity of competitive outcomes as classification criteria. In youth tennis, birthdate has been found to influence physical test outcomes at U12 level [22,23]; at U10, where a twelve-month age gap represents a greater fraction of total developmental experience, competitive rankings are particularly susceptible to the Relative Age Effect (RAE) and potentially unreliable as indicators of underlying talent. Imposing such rankings as external classification labels risks introducing systematic bias into any criterion-based analytical model. Cluster analysis offers a preferable alternative by identifying internally coherent groups based on the multidimensional structure of the data, without assuming that early rankings reflect true ability or imposing constraints on developmental heterogeneity [3].

Against this background, the present study used an integrated, purpose-designed test battery to assess physical-motor performance, technical skill execution, and reaction-based cognitive performance in 113 players enrolled in a national talent identification programme. The study offers a dual contribution: methodologically, it combines these domains within a single profiling framework not previously applied at the U10 level; practically, it provides a foundation for more individualised performance profiling and evidence-informed player development during the pre-PHV stage.

Given the exploratory nature of the present study and the absence of predefined performance categories, no directional hypotheses regarding the number or characteristics of the resulting profiles were formulated. Rather, we expected that integrating technical, physical-motor, and reaction-based cognitive measures within a data-driven analytical framework would reveal meaningful multidimensional heterogeneity among U10 tennis players. Accordingly, the present study aimed to identify and characterise naturally occurring multidimensional performance profiles without assuming a priori cluster membership or ordering.

## 2. Materials and Methods

### 2.1. Participants

A total of 113 youth tennis players (73 boys and 40 girls, mean age = 9.06 ± 0.80 years) participating in the Hungarian Tennis Federation’s U10 Talent Identification Festival were included in this cross-sectional study. Participation in the festival was open to all players belonging to the U10 age category; no performance-based, ranking-based, or federation-squad eligibility criteria were applied. For the present retrospective analysis, participants were included if they took part in the festival and had complete data for the performance variables included in the multivariate analyses. No additional performance-based exclusion criteria were applied. During data verification, two standing long jump values initially recorded as 0 cm in the electronic dataset were identified as data-entry errors. The corresponding original paper-based assessment records were retrieved, and the verified measured values were entered into the analytical dataset before the statistical analyses were rerun. No missing performance data remained in the final analytical sample (N = 113).

Data collection took place on 16 May 2026 at the National Training Centre in Budapest, Hungary. The event formed part of the Hungarian Tennis Federation’s national talent identification programme.

### 2.2. Testing Procedure

Assessments were conducted using a station-based format during the Hungarian Tennis Federation U10 Talent Identification Festival. Participants progressed through the testing stations in a fixed order. A brief task-specific warm-up was performed before each station. Approximately 5 min of preparation was provided before the sprint assessment, including running drills and progressive movement preparation, whereas approximately 2 min of task-specific preparation was used at the remaining stations. Warm-up activities were adapted to the demands of the subsequent assessment and included running drills, dynamic mobility exercises, and upper-body/shoulder mobilisation where appropriate. Familiarisation with unfamiliar tasks was provided before recorded trials. Identical station order, task instructions, and scoring procedures were applied across participants.

### 2.3. Ethics Statement

The study was conducted in accordance with the Declaration of Helsinki and was approved by the Research Ethics Committee of the Hungarian University of Sports Science (Approval No. MTSE-KEB/No21/2025). Performance data were originally collected as part of the Hungarian Tennis Federation’s U10 Talent Identification Programme rather than specifically for the present research analysis. Parents or legal guardians had provided consent for their children’s participation in the programme and for the publication of their performance results by the Federation. For the present retrospective research analysis, the data were anonymized prior to statistical processing, and no personally identifiable information was included in the research dataset. The retrospective research use of the anonymized programme data was covered by the ethical approval granted by the Research Ethics Committee of the Hungarian University of Sports Science (Approval No. MTSE-KEB/No21/2025).

### 2.4. Study Design

This cross-sectional study was conducted during the Hungarian Tennis Federation’s U10 Talent Identification Festival in 2026 at the National Training Centre in Hungary. The purpose of the event was to evaluate the multidimensional performance characteristics of young tennis players within a standardised testing environment.

Participants completed an integrated test battery consisting of ten assessment stations. The battery was specifically designed to capture complementary domains of early tennis performance, including technical skills, physical-motor abilities, reaction-based cognitive-perceptual performance, and selected neuromuscular characteristics. All assessments were administered by trained coaches and sport science staff following standardised testing procedures. Performance at each station was recorded immediately after completion using predefined scoring criteria.

The resulting dataset provided a multidimensional representation of each player’s performance profile and formed the basis for the subsequent cluster analysis.

### 2.5. Test Battery

The assessment battery was developed for applied use within the Hungarian Tennis Federation’s U10 Talent Identification Programme through expert consensus among federation coaches and practitioners. The battery was designed to capture complementary technical, physical-motor, and reaction-based performance characteristics relevant to early tennis development. It was developed as an applied field-assessment framework rather than as a formally validated research instrument, and formal test–retest reliability, inter-rater reliability, and construct validity have not yet been established for the complete battery. The multidimensional test battery consisted of ten assessment stations designed to evaluate complementary domains of tennis performance. Each station targeted a specific technical, physical-motor, or reaction-based cognitive-perceptual competency considered relevant for early tennis development. A brief description of each assessment is provided below. Before testing, all coaches and sport science staff received standardized instructions regarding task administration, scoring procedures, and recording of performance outcomes. Throughout the festival, identical protocols and predefined scoring criteria were applied across all testing stations to maximize procedural consistency.

### 2.6. Serve Accuracy Assessment

Players performed ten serves from the deuce court, aiming at numbered target cones positioned within the service box. Each serve landing within the service box received 1 point. If the serve also struck a designated target, the numerical value displayed on that target was added to the score for that serve. The accumulated score across the ten serves was retained for analysis.

### 2.7. Volley Skill Assessment

Players performed 20 volleys while moving toward balls hand-fed alternately to the forehand and backhand sides by an assessor. The first ten trials required deep volleys, whereas the second ten required drop volleys. For deep volleys, 5 points were awarded when the ball first bounced between the service line and baseline, and 10 points when it struck the designated rear target area. For drop volleys, 5 points were awarded when the ball first bounced between the net and service line, and 10 points when it struck the designated target area immediately behind the net. The accumulated score across the 20 trials was retained for analysis.

### 2.8. Topspin and Slice Execution Assessment

Players performed 20 groundstrokes from balls hand-fed alternately to the forehand and backhand sides by an assessor. During the first ten strokes, players were required to produce topspin from both the forehand and backhand sides, with the ball passing above a red rope suspended above the net. During the subsequent ten strokes, players were required to produce slice from both sides, with the ball passing through the space between the net and the red rope. A correctly executed stroke that passed through the required trajectory and first bounced within the court received 1 point; a ball first bouncing in the designated deep target zone between the service line and baseline received 5 points; and a ball additionally striking a designated target received 10 points. The accumulated score across the 20 trials was retained for analysis.

### 2.9. Groundstroke Accuracy Assessment

Players performed ten strokes directed toward a target board. A successful hit on the board received 5 points. If the ball additionally entered one of the numbered target openings, the numerical value assigned to that opening was added to the 5-point base score. The accumulated score across the ten trials was retained for analysis.

### 2.10. Grip Strength Assessment

Maximal handgrip strength was assessed using an ER101 digital hand dynamometer. Participants performed two maximal trials with each hand while standing with the tested arm extended. Trials alternated between the right and left hands, thereby providing a brief recovery period between repeated attempts with the same hand. The highest value (kg) recorded across the four trials was retained for subsequent analyses. The manufacturer and detailed measurement characteristics of the ER101 device could not be reliably recovered from the historical testing documentation.

### 2.11. Standing Long Jump Assessment

Lower-body explosive power was assessed using the standing long jump test. Participants performed two maximal jumps from a standing start, and the longest jump distance (cm) was recorded for analysis.

### 2.12. BlazePod Reaction Assessment

Reaction-based performance was assessed using a BlazePod system comprising four light-based pods positioned on four cones. Participants completed two 30-s tasks. In the first task, they were instructed to respond as quickly as possible by tapping each pod whenever it illuminated. In the second task, participants were required to respond selectively, tapping only the pod displaying a predefined target colour while withholding responses to other colours. The aim in both tasks was to achieve as many correct responses as possible within the 30-s period. The total number of successful responses across the two tasks was summed and retained as the reaction-performance score for analysis. The task therefore primarily assessed rapid visuomotor responding and selective attention under generic stimulus–response conditions and should not be interpreted as a direct measure of tennis-specific anticipation or decision-making.

### 2.13. Ball Throwing Velocity Assessment

Upper-body throwing performance was assessed using a radar-based ball-velocity task conducted at one of the testing stations provided for the programme by BlazePod. Participants performed an overarm throw, using a throwing action comparable to that typically employed in handball, toward a vertically positioned target net from a distance of three metres. Ball velocity was recorded in km·h^−1^. Two maximal trials were performed, and the highest recorded velocity was retained for analysis. As the assessments were originally conducted as part of an applied Federation talent-identification event rather than a prospectively designed research protocol, the specific manufacturer and model of the radar device were not retained in the historical testing documentation.

### 2.14. 15-m Sprint Assessment

Linear sprint performance was assessed over 15 m using OxaSport photocell timing gates (OXA Sport Timer Bt., Ócsa, Hungary). Participants started from a standing position approximately 0.4 m behind the first timing gate to minimise inadvertent triggering of the photocell by forward body or arm movement. Two maximal trials were completed, with approximately 5 min of recovery between trials while the remaining participants completed their attempts. The faster sprint time (s) was retained for subsequent analyses.

### 2.15. Agility Multiskill Assessment

Agility and general movement coordination were evaluated using a standardised multiskill obstacle course comprising slalom running, ball-control tasks, climbing on wall bars, and jumping tasks. Participants were instructed to complete the course as quickly as possible while correctly performing each component. If an obstacle was completed incorrectly, the participant was required to repeat that component before continuing. Total completion time (s) represented performance.

A detailed description of the assessment battery is provided in Table 1.

### 2.16. Statistical Analysis

Descriptive statistics are presented as means ± standard deviations. No a priori power analysis was conducted because the primary analyses were exploratory PCA and cluster analysis rather than hypothesis-driven inferential tests. The final analytical sample included 113 participants across 10 performance variables, corresponding to a participant-to-variable ratio of approximately 11:1. Sampling adequacy was additionally evaluated empirically using the Kaiser–Meyer–Olkin statistic and Bartlett’s test of sphericity. Prior to multivariate analyses, all performance variables were standardized using z-scores to eliminate differences in measurement scales. Principal component analysis (PCA) was performed on the standardized performance variables as a complementary examination of the correlation structure and dimensional characteristics of the assessment battery.

Sampling adequacy was evaluated using the Kaiser–Meyer–Olkin (KMO) measure, and Bartlett’s test of sphericity was used to assess whether the correlation matrix was suitable for PCA. Component retention was based on parallel analysis with 10,000 Monte Carlo replications (random seed = 20,260,816) and the 95th-percentile random-eigenvalue criterion. Independent normally distributed data were used to derive the retention thresholds, while column-wise resampling with replacement of the observed data provided an additional reference. Numerical PCA outputs, including eigenvalues, explained variance, and component loadings, are reported to improve transparency of the PCA procedure.

The optimal number of clusters was evaluated using three complementary criteria: the elbow method, average silhouette width, and the gap statistic. Because these indices provided partially divergent indications, both two- and three-cluster solutions were examined further. Cluster quality was compared using average silhouette width, within-cluster sum of squares, and the proportion of total variance attributable to between-cluster differences. Cluster stability was additionally evaluated using bootstrap resampling and mean Jaccard similarity coefficients. Considering the quantitative validation indices, bootstrap stability, and parsimony, the two-cluster solution was retained as the primary exploratory representation of multidimensional performance heterogeneity.

K-means clustering was performed using the 10 standardized performance variables. The resulting clusters were subsequently characterised according to their multidimensional performance profiles across the technical, physical-motor, and reaction-based measures.

Between-cluster differences in performance variables were evaluated using Welch’s independent-samples *t*-tests, which were selected because several variables showed evidence of non-normality and/or unequal variances. Effect sizes were quantified using Hedges’ *g* with 95% confidence intervals. For variables showing pronounced departures from distributional assumptions, Wilcoxon rank-sum tests were additionally performed as sensitivity analyses. Differences in chronological age between clusters were assessed using Welch’s independent-samples *t*-test, with Hedges’ *g* reported as an effect-size estimate. Associations between cluster membership and sex or birth quarter were examined using chi-square tests. Pearson’s chi-square test was used for birth quarter, whereas Yates’ continuity correction was applied to the 2 × 2 sex table. Statistical significance was established at *p* < 0.05.

To examine the potential influence of chronological age on the clustering solution, an age-adjusted sensitivity analysis was conducted. For each performance variable, the linear effect of chronological age was removed and the resulting residuals were standardized before repeating the k-means clustering procedure with two clusters. Agreement between the original and age-adjusted cluster assignments was examined descriptively, and the stability of the age-adjusted solution was evaluated using bootstrap resampling and mean Jaccard similarity coefficients. Average silhouette width was also calculated for the age-adjusted clustering solution.

Statistical analyses were performed using JASP (Version 0.19.3.0; JASP Team, Amsterdam, The Netherlands) and R (Version 4.6.1; R Foundation for Statistical Computing, Vienna, Austria). Principal component analysis, cluster analysis, and data visualization were conducted in R using the psych, FactoMineR, factoextra, cluster, and ggplot2 packages.

The clustering procedure was treated as an exploratory multivariate analysis intended to identify naturally occurring performance groupings rather than predefined performance categories.

Principal component loadings and additional cluster-validation results are provided as Supplementary Material to improve transparency and reproducibility.

## 3. Results

### 3.1. Descriptive Statistics

Descriptive statistics for the final analytical sample (N = 113) are presented in Table 2. No missing values remained after data cleaning.

### 3.2. Principal Component Analysis

Principal component analysis (PCA) was performed on the standardized performance variables to examine the dimensional structure of the assessment battery. The Kaiser–Meyer–Olkin measure indicated good sampling adequacy (KMO = 0.81), and Bartlett’s test of sphericity was significant, χ^2^(45) = 266.80, *p* < 0.001, supporting the suitability of the correlation matrix for PCA. Parallel analysis supported the retention of one principal component. The first principal component had an eigenvalue of 3.620 and accounted for 36.20% of the total variance. The second observed eigenvalue (1.276) was below the corresponding 95th-percentile threshold from the simulated data (1.436) and therefore did not meet the parallel-analysis retention criterion.

**Figure 1:**
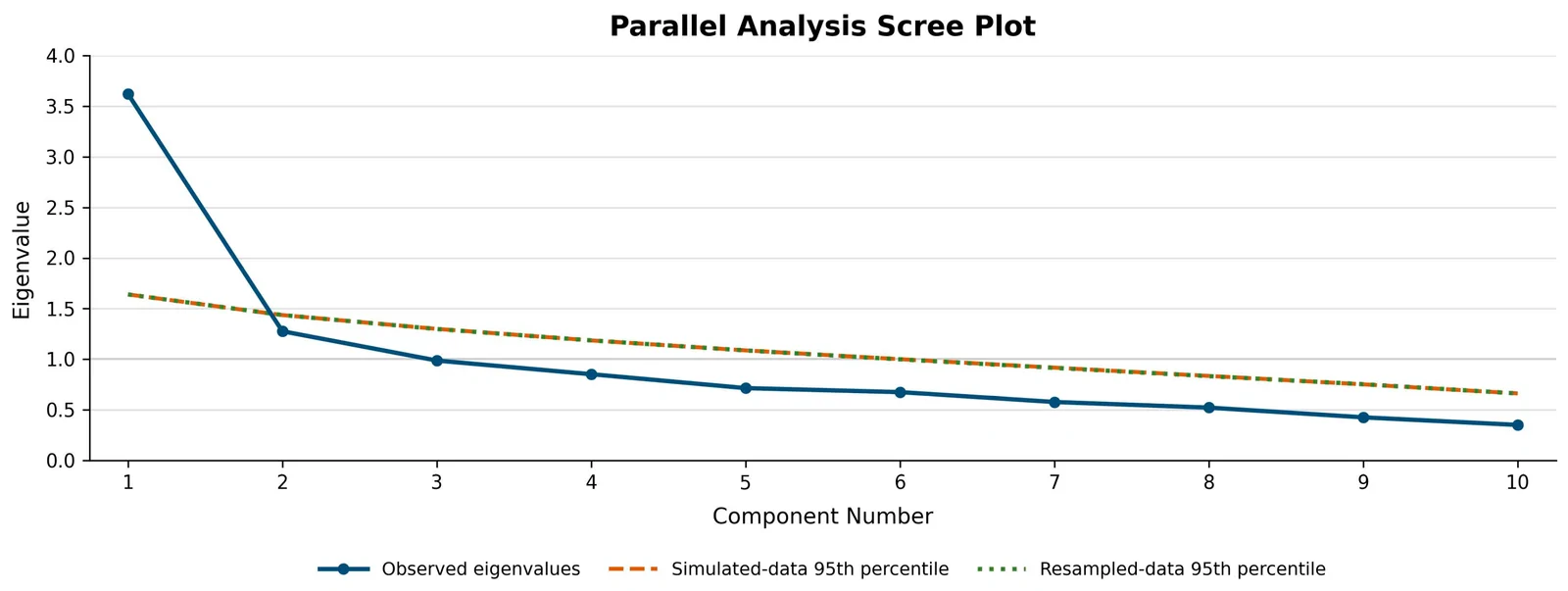
Parallel analysis scree plot for principal component retention. Observed eigenvalues are compared with 95th-percentile thresholds from 10,000 replications of independent normally distributed simulated data and column-wise resampled observed data. Parallel analysis supported the retention of one principal component.

As illustrated in Figure 1, only the first observed eigenvalue exceeded the corresponding 95th-percentile thresholds derived from both simulated and resampled data, supporting the retention of a single principal component.

### 3.3. Cluster Validation

The complementary cluster-validation criteria provided partially divergent indications regarding the optimal number of clusters. Average silhouette width favoured the two-cluster solution (0.211) over the three-cluster solution (0.141), whereas the gap statistic favoured three clusters. The elbow criterion indicated diminishing improvements in within-cluster compactness as the number of clusters increased. Total within-cluster sum of squares decreased from 844.98 for the two-cluster solution to 749.78 for the three-cluster solution, while the proportion of total variance attributable to between-cluster differences increased from 24.6% to 33.1%. Bootstrap resampling nevertheless indicated high stability of the two-cluster solution, with mean Jaccard similarity coefficients of 0.925 and 0.947 for the two clusters. Considering the higher average silhouette width, high bootstrap stability, and greater parsimony, the two-cluster solution was retained for the primary analyses.

**Figure 2:**
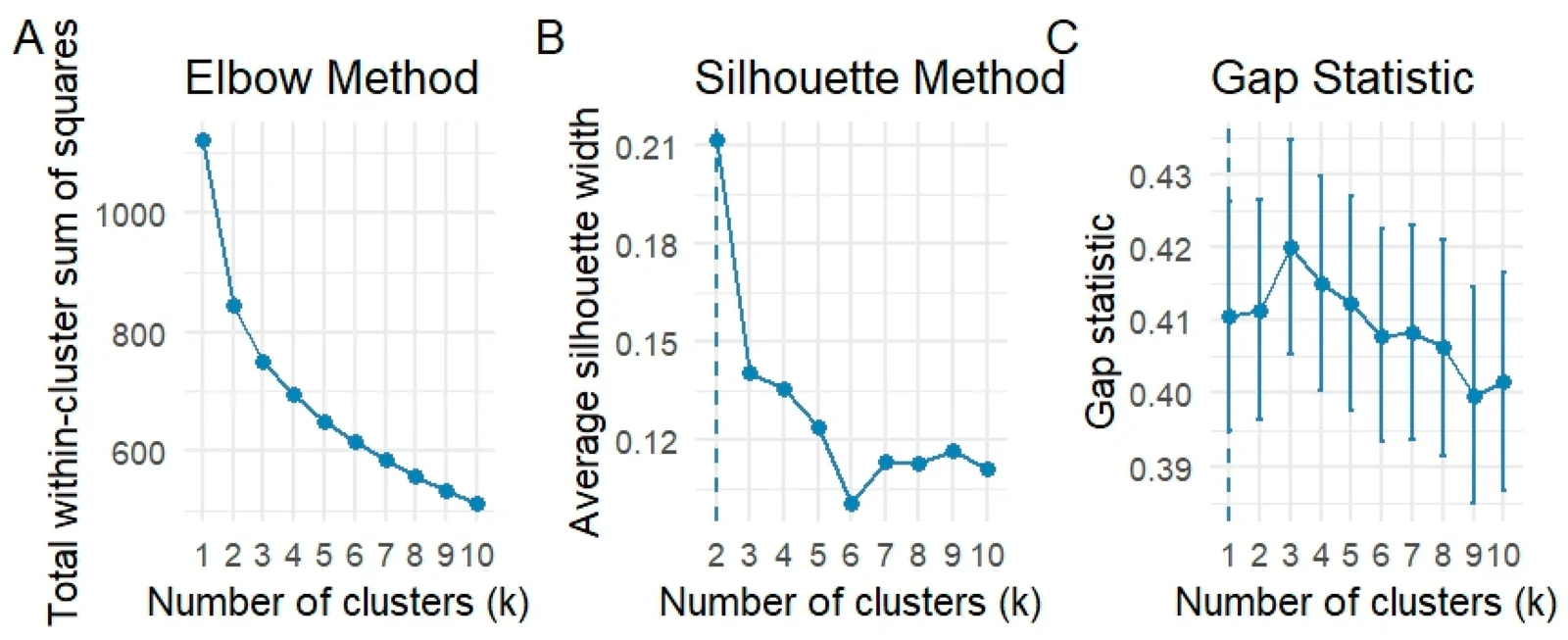
Cluster-validation procedures used to evaluate the optimal number of clusters. (**A**) Elbow method based on total within-cluster sum of squares. (**B**) Average silhouette width across candidate cluster solutions. (**C**) Gap statistic across candidate cluster solutions. The validation criteria provided partially divergent indications: average silhouette width favoured the two-cluster solution, whereas the gap statistic favoured three clusters. Considering the higher silhouette width of the two-cluster solution, its high bootstrap stability, and greater parsimony, two clusters were retained for the primary analyses.

The results of the elbow, silhouette, and gap-statistic procedures are presented in Figure 2.

### 3.4. Cluster Analysis

**Figure 3:**
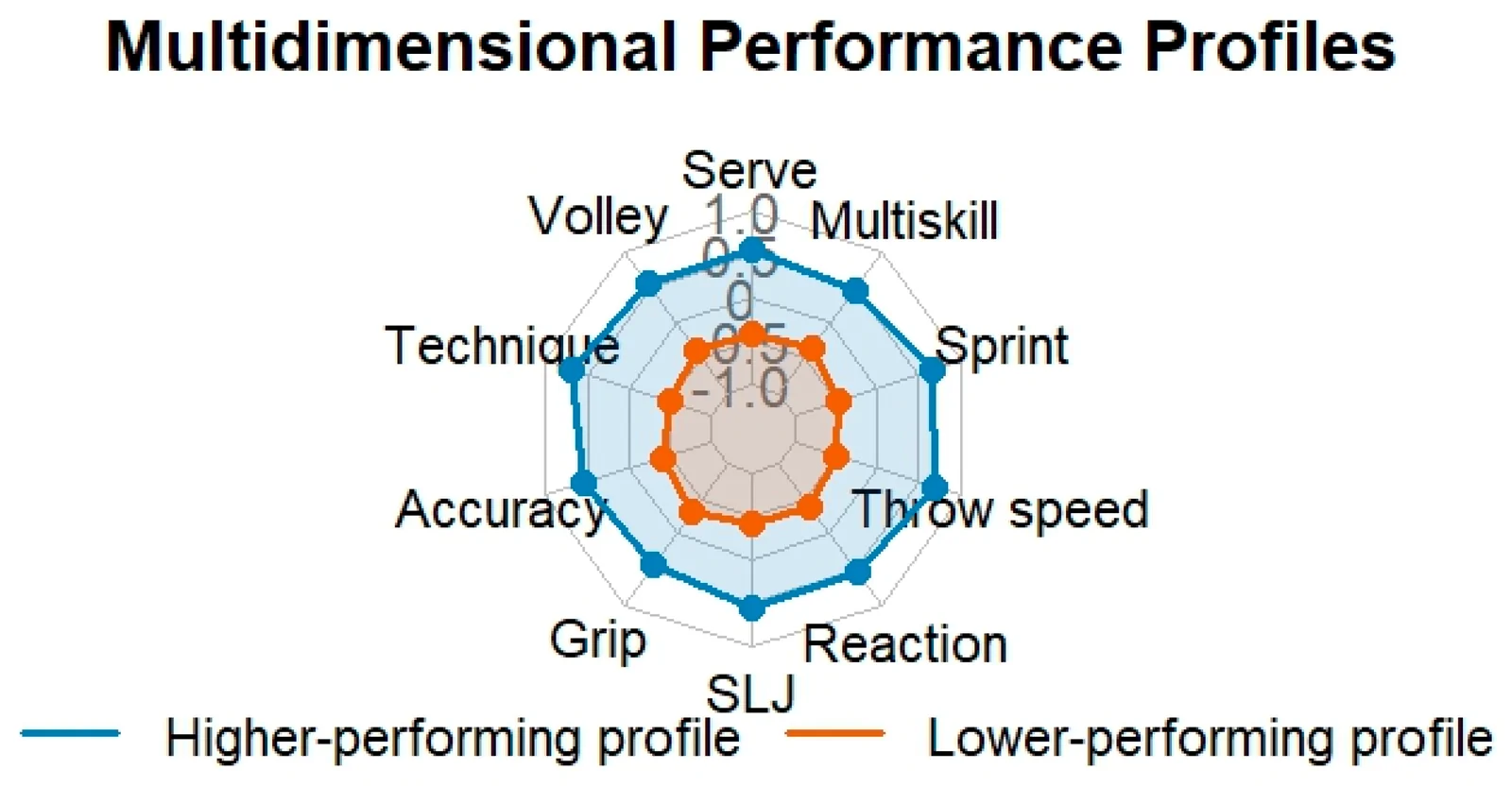
Standardized multidimensional performance profiles of the two-cluster solution. Values represent standardized cluster centroids (z-scores). For the 15-m sprint and multiskill course, signs were reversed for visualization so that higher values consistently indicate better performance across all dimensions.

The final two-cluster solution comprised 48 participants in Cluster 1 and 65 participants in Cluster 2. Examination of the standardized cluster centroids indicated a consistent overall performance gradient across the multidimensional assessment battery. Cluster 1 showed higher standardized scores for serve, volley, technique, accuracy, grip strength, standing long jump, reaction performance, and throwing speed, together with lower sprint and multiskill completion times, indicating better performance across all assessed domains. Cluster 2 showed the opposite pattern. Accordingly, the clusters are hereafter referred to descriptively as the higher-performing profile (n = 48) and lower-performing profile (n = 65), rather than as talent categories (Figure 3).

### 3.5. Cluster Characteristics

Mean values for all technical and physical performance variables within each performance profile are presented in Table 3.

The two-cluster solution differentiated a higher-performing profile (n = 48) from a lower-performing profile (n = 65). The higher-performing profile demonstrated better mean performance across all assessed technical, physical-motor, and reaction-based measures. The largest standardized differences were observed for throwing speed (Hedges’ g = 1.43), technique (g = 1.42), and 15-m sprint performance (g = −1.38), while substantial differences were also observed across the remaining performance variables (|g| = 0.81–1.10). These findings indicate broad multidimensional separation between the two profiles rather than differentiation driven by a single performance domain. However, because cluster membership was also associated with chronological age, the profiles should be interpreted as descriptive performance groupings within the present sample rather than as distinct talent categories.

Welch’s independent-samples *t*-tests indicated significant differences between the higher- and lower-performing profiles across all ten performance variables (all *p* < 0.001). Effect sizes were large across all comparisons, with absolute Hedges’ g values ranging from 0.81 for grip strength to 1.43 for throwing speed. Detailed test statistics and 95% confidence intervals are presented in Table 4. Because the same performance variables were used to derive the cluster solution, these between-profile comparisons are presented for descriptive characterisation of the identified profiles and should not be interpreted as independent inferential validation of the cluster structure.

Chronological age differed significantly between the two performance profiles. Participants in the higher-performing profile were older than those in the lower-performing profile (9.41 vs. 8.80 years; Welch’s t(102.94) = 4.24, *p* < 0.001; Hedges’ g = 0.80, 95% CI [0.41, 1.18]). Given the magnitude of this difference, an age-adjusted sensitivity analysis was conducted to examine whether the observed cluster structure was primarily attributable to chronological age. After residualising each performance variable for age and repeating the two-cluster analysis, the age-adjusted solution remained highly stable, with bootstrap mean Jaccard coefficients of 0.937 and 0.946. Overall, 96 of 113 participants (85.0%) retained corresponding cluster membership. Average silhouette width decreased from 0.211 in the original solution to 0.184 after age adjustment. These findings indicate that chronological age contributed to the observed performance grouping but did not fully account for the two-cluster structure.

Birth-quarter distribution was not significantly associated with performance-profile membership, χ^2^(3) = 0.28, *p* = 0.964. The distribution of participants across birth quarters within the two profiles is presented in Table 5.

Sex was not significantly associated with performance-profile membership, χ^2^(1) = 3.19, *p* = 0.074 (Table 6).

**Table 6:** Sex distribution across the two performance profiles.

Sex	Higher-Performing Profile (n = 48)	Lower-Performing Profile (n = 65)
Female	12	28
Male	36	37

Note. The chi-square test with Yates’ continuity correction indicated no statistically significant association between sex and performance-profile membership, χ^2^(1) = 3.19, *p* = 0.074.

## 4. Discussion

The present study aimed to identify multidimensional performance profiles in U10 tennis players using an integrated assessment battery encompassing technical, physical-motor, and reaction-based cognitive-perceptual performance domains. The primary analysis identified two stable performance profiles, characterised by consistently higher and lower performance across the assessed dimensions. Although the higher-performing profile demonstrated better performance across all ten variables, the magnitude of between-profile differences varied across individual measures, indicating that the separation was multidimensional rather than attributable to a single performance characteristic. Importantly, chronological age differed significantly between the profiles. However, the two-cluster structure remained highly stable after age adjustment, indicating that age contributed to, but did not fully account for, the observed performance grouping. Accordingly, these profiles should not be interpreted as fixed talent categories or developmental trajectories, but rather as data-driven descriptions of current multidimensional performance within the present sample.

The identification of multidimensional performance profiles has important implications for early talent identification in tennis. Traditional talent identification programmes have frequently relied on isolated performance indicators or competitive rankings to evaluate young athletes [1,4]. However, accumulating evidence suggests that athletic development is inherently heterogeneous, with successful athletes following multiple developmental trajectories rather than a single linear pathway [3,5]. In the present study, the two profiles were characterised by a consistent overall performance gradient, with the higher-performing profile demonstrating better performance across all assessed technical, physical-motor, and reaction-based measures. Nevertheless, the magnitude of between-profile differences varied across individual variables, with particularly large differences in throwing speed, technique, and sprint performance. Thus, the multidimensional approach provided information not only about overall performance level but also about the relative magnitude of separation across specific performance characteristics. Such profiling may therefore offer a more comprehensive description of current performance heterogeneity than reliance on any single test or competitive ranking.

The present findings also reinforce the importance of integrating multiple performance domains within talent identification programmes. Previous research has consistently demonstrated that physical characteristics such as sprint speed, explosive power, agility, and throwing performance contribute substantially to youth tennis performance [2,7,8]. Likewise, technical proficiency has been recognised as a fundamental determinant of competitive success, yet it has often been assessed using simplified outcome measures that fail to capture the multidimensional nature of stroke execution [11,12]. More recently, Oliveira et al. [13] highlighted the need for multidimensional approaches capable of identifying distinct performance characteristics among young tennis players. Evidence from youth soccer similarly indicates interrelationships among physical fitness, motor coordination, and sport-specific technical skills, further supporting integrated assessment [24]. The present study extends this body of evidence by showing that combining technical and physical-motor measures with a reaction-based cognitive-perceptual measure within a single analytical framework allows the identification of distinct multidimensional performance profiles that would be difficult to detect using isolated performance measures alone.

The multidimensional performance groupings identified in the present study likely reflect the complex interaction of biological, training-related, and individual developmental factors that characterise early childhood sport participation. This interpretation is consistent with recent evidence showing that biological maturation and training experience influence motor performance differently from perceptual-cognitive performance, suggesting that these domains may follow partially independent developmental pathways [25]. Although biological maturation is generally less influential before peak height velocity than during adolescence [20,21], children of the same chronological age may still differ considerably in training experience, motor coordination, technical instruction, practice volume, and learning opportunities. Such differences may contribute to the emergence of distinct performance profiles despite relatively small age differences. Because technical skills, physical-motor capacities, and reaction-based performance may not develop at identical rates during late childhood, evaluating these domains simultaneously may provide a more informative description of current developmental status than relying on isolated physical tests or competitive outcomes alone.

An additional finding of particular interest was the absence of an association between birth quarter and performance-profile membership. Relative Age Effects (RAEs) have been consistently reported across a wide range of youth sports, including tennis, where relatively older athletes may demonstrate advantages in physical performance and selection into development programmes [22,23]. Based on this evidence, a greater representation of relatively older players might have been expected within the higher-performing profile. However, birth-quarter distribution did not differ between the two profiles in the present sample. These findings suggest that birth quarter alone did not explain membership in the identified multidimensional performance profiles. Importantly, this absence of a birth-quarter association should be distinguished from the significant difference in chronological age observed between the profiles, as birth quarter and exact chronological age capture related but non-equivalent aspects of age variation within an age-group category.

Chronological age differed significantly between the two performance profiles, indicating that age-related developmental differences contributed meaningfully to the observed performance grouping. However, the age-adjusted sensitivity analysis provided important additional context: after accounting for chronological age, the two-profile structure remained highly stable and showed substantial correspondence with the original cluster solution. This persistence indicates that chronological age contributed to, but did not fully account for, the observed performance heterogeneity. Accordingly, the profiles should be interpreted as descriptions of current multidimensional performance that are partly influenced by age-related development, rather than as either age-independent indicators of underlying talent or simple reflections of chronological age.

Sex was not significantly associated with performance-profile membership in the present sample. Although boys represented a larger proportion of the higher-performing profile, the association did not reach statistical significance. Accordingly, the observed distribution should not be interpreted as evidence of sex-specific differences in multidimensional performance profiles. Future studies with larger and more balanced samples should examine whether sex contributes to performance-profile membership after accounting for chronological age, training age, and biological maturation.

From an applied perspective, multidimensional profiling may provide coaches with a structured way of interpreting young players’ current performance characteristics across several domains simultaneously. In the present sample, the two profiles primarily reflected a broad performance gradient rather than sharply contrasting domain-specific patterns, with the higher-performing profile demonstrating better mean performance across all assessed variables. Nevertheless, the magnitude of between-profile differences varied across measures, which may help identify performance characteristics that most strongly differentiate players within this developmental context. Such information may complement, rather than replace, longitudinal observation, coach judgement, competitive performance, and individual developmental monitoring. Importantly, cluster membership should not be used as a selection or deselection criterion. The present profiles are descriptive and cross-sectional, and their longitudinal stability and predictive validity for later tennis performance remain unknown.

### Strengths and Limitations

The present study has several important strengths. To our knowledge, it is the first investigation to apply a multidimensional profiling approach to U10 tennis players by integrating technical and physical-motor measures with a reaction-based cognitive-perceptual measure within a single analytical framework. In addition, the study was conducted within the Hungarian Tennis Federation’s national talent identification programme, providing a relatively large sample of competitively active young players assessed under standardized field conditions. PCA provided a complementary examination of the battery’s correlation structure, while k-means clustering of the ten standardized performance variables enabled data-driven examination of performance heterogeneity without relying on a single performance indicator or competitive ranking. Cluster-number selection was evaluated using multiple complementary criteria, and bootstrap resampling was used to assess the stability of the retained solution. Furthermore, the age-adjusted sensitivity analysis provided an additional test of whether the observed grouping could be explained primarily by chronological age.

Several limitations should also be acknowledged. First, the cross-sectional design does not allow conclusions regarding the longitudinal stability of the identified performance profiles or their ability to predict future competitive success. Longitudinal follow-up is therefore required to determine whether these profiles persist over time and whether profile membership is associated with subsequent athletic development. Second, chronological age differed significantly between the two profiles, indicating that age-related development contributed to the observed performance grouping. Although the age-adjusted sensitivity analysis showed that the two-profile structure remained highly stable after controlling for chronological age, age cannot be considered fully separable from performance development in this cross-sectional sample. Moreover, biological maturation and training age were not assessed and may account for additional developmental variability between players. Third, the study was conducted within a single national talent identification programme, which may limit the generalisability of the findings to other countries, competitive levels, or development systems. Finally, although the assessment battery incorporated multiple performance domains, psychological and tactical variables were not included and may provide additional information in future multidimensional profiling studies.

The assessment battery was developed for applied use within the Hungarian Tennis Federation’s U10 Talent Identification Programme rather than as a formally validated research instrument. Although it differentiated the two performance profiles in the present sample, its psychometric properties have not yet been established. In particular, test–retest reliability, inter-rater reliability for the technical assessments, and construct validity require formal evaluation. Consequently, measurement error and assessor-related variability cannot be excluded and should be considered when interpreting the identified performance profiles.

Although the Kaiser–Meyer–Olkin statistic indicated good sampling adequacy (KMO = 0.81), the participant-to-variable ratio remained relatively modest for PCA. Moreover, parallel analysis supported retention of a single principal component, suggesting that the assessed performance variables were characterised predominantly by a common underlying performance dimension rather than multiple clearly separable latent dimensions. The PCA should therefore be interpreted as a complementary examination of the battery’s dimensional structure rather than as evidence of multiple stable latent dimensions; the subsequent k-means clustering was performed independently on the ten standardized performance variables. Replication in larger independent samples is warranted to evaluate the reproducibility of both the component structure and the performance profiles.

## 5. Conclusions

The present findings support multidimensional profiling as a feasible exploratory framework for characterising performance heterogeneity in U10 tennis players. The retained two-profile solution differentiated players with consistently higher and lower performance across technical, physical-motor, and reaction-based cognitive-perceptual measures, although the magnitude of between-profile differences varied across individual performance variables.

Chronological age was significantly associated with profile membership, with players in the higher-performing profile being older on average. However, the persistence and high bootstrap stability of the two-profile solution after age adjustment indicate that chronological age contributed to, but did not fully account for, the observed performance grouping. In contrast, neither birth quarter nor sex was significantly associated with profile membership in the present sample.

These findings suggest that multidimensional assessment may complement traditional approaches based on isolated performance measures or competitive rankings by providing a broader description of a young player’s current performance status. In practice, coaches may use such assessments to identify relative strengths and development needs across technical, physical-motor, and reaction-based domains, rather than basing developmental decisions on a single test or competitive outcome. Repeated multidimensional assessment may also help coaches monitor how individual performance characteristics evolve over time and support the prioritisation of training content according to each player’s current profile. Importantly, chronological age and other developmental factors should be considered when interpreting performance differences between U10 players. The identified profiles should not be interpreted as fixed talent categories or used as stand-alone selection or deselection criteria. Given the cross-sectional design, the absence of biological maturation and training-age measures, and the currently unestablished predictive validity of the profiles, longitudinal research is required to determine whether these performance groupings remain stable over time and whether they are associated with subsequent tennis development and competitive success.

## Figures and Tables

**Table 1:** Overview of the multidimensional performance assessment battery.

Assessment	Performance Domain	Primary Competency Assessed	Outcome Variable
Serve accuracy	Technical	Serve accuracy and ball placement	Total score
Groundstroke accuracy	Technical	Stroke accuracy	Total score
Topspin and slice execution	Technical	Stroke technique and ball control	Total score
Volley skill	Technical	Volley control and touch	Total score
Grip strength	Physical-motor	Maximal grip strength	kg
Standing long jump	Physical-motor	Lower-body explosive power	cm
Ball throwing velocity	Physical-motor	Upper-body explosive power	km·h^−1^
Agility multiskill course	Physical-motor	Agility and movement coordination	s
15-m sprint	Physical-motor	Linear sprint performance	s
BlazePod reaction test	Reaction-based cognitive-perceptual	Reaction speed and selective attention	Total touches

Note. The assessment battery was designed to capture complementary dimensions of tennis performance relevant to early talent identification, including technical, physical-motor, and reaction-based cognitive-perceptual characteristics.

**Table 2:** Descriptive statistics of participant characteristics and performance variables.

Variable	Mean ± SD	Minimum	Maximum
Age (years)	9.06 ± 0.80	6.95	10.37
Serve (points)	13.08 ± 8.93	0	43
Volley (points)	70.62 ± 21.96	10	120
Technique (points)	31.27 ± 17.77	2	89
Accuracy (points)	133.01 ± 77.73	15	320
Grip (kg)	18.05 ± 3.97	8.70	38.10
SLJ (cm)	154.12 ± 14.88	113	193
Reaction (hits)	73.39 ± 8.51	56	104
Throw speed (km/h)	53.48 ± 11.42	27	80
Sprint15 (s)	2.99 ± 0.18	2.66	3.47
Multiskill (s)	41.26 ± 10.35	25.59	95.31

**Table 3:** Mean ± SD technical, physical-motor, and reaction-based cognitive performance characteristics of the two identified performance profiles.

Variable	Higher-Performing Profile (n = 48)	Lower-Performing Profile (n = 65)
Serve (points)	18.0 ± 9.46	9.46 ± 6.52
Volley (points)	82.8 ± 20.7	61.6 ± 18.4
Technique (points)	43.5 ± 17.2	22.3 ± 11.9
Accuracy (points)	176 ± 88.9	101 ± 48.4
Grip strength (kg)	19.8 ± 3.83	16.8 ± 3.58
Standing long jump (cm)	162 ± 12.6	148 ± 13.5
Reaction (hits)	78.0 ± 7.81	70.0 ± 7.38
Throwing speed (km·h^−1^)	61.3 ± 10.3	47.7 ± 8.42
15-m sprint (s)	2.87 ± 0.125	3.07 ± 0.163
Multiskill test (s)	36.4 ± 6.20	44.9 ± 11.3

**Table 4:** Welch’s independent-samples *t*-tests and effect sizes for differences between the higher- and lower-performing profiles.

Variable	Welch t	df	*p*	Hedges’ g	95% CI
Serve	5.37	78.68	<0.001	1.04	[0.62, 1.45]
Volley	5.65	94.26	<0.001	1.08	[0.67, 1.48]
Technique	7.35	78.97	<0.001	1.42	[0.98, 1.86]
Accuracy	5.29	67.41	<0.001	1.04	[0.61, 1.45]
Grip strength	4.28	97.52	<0.001	0.81	[0.42, 1.20]
Standing long jump	5.84	104.87	<0.001	1.10	[0.70, 1.49]
Reaction	5.49	98.11	<0.001	1.04	[0.64, 1.44]
Throwing speed	7.45	88.81	<0.001	1.43	[0.99, 1.85]
15-m sprint	−7.45	110.84	<0.001	−1.38	[−1.78, −0.97]
Multiskill	−5.09	103.29	<0.001	−0.92	[−1.30, −0.54]

Note. Hedges’ *g* represents the standardized difference between the higher-performing and lower-performing profiles. Negative effect sizes for the 15-m sprint and multiskill test reflect better performance through shorter completion times in the higher-performing profile. CI = confidence interval.

**Table 5:** Distribution of birth quarters across the two performance profiles.

Birth Quarter	Higher-Performing Profile (n = 48)	Lower-Performing Profile (n = 65)
Q1	18	22
Q2	12	16
Q3	8	13
Q4	10	14

## Data Availability

The performance data analysed in the present study were collected as part of the Hungarian Tennis Federation’s U10 Talent Identification Programme. The performance results are also publicly accessible through the official website of the Hungarian Tennis Federation. Additional processed data supporting the findings of this study are available from the corresponding author upon reasonable request.

## Supplementary Materials

The following supporting information can be downloaded at: https://www.mdpi.com/article/10.3390/sports14090382/s1, Supplementary Table S1: Principal component loadings for the retained first principal component.
