# Supplementary material for: Data-Driven Multidimensional Performance Profiling in U10 Tennis Players
Source: Sports (Basel). 2026 Sep 1;14(9):382. doi: 10.3390/sports14090382 (PMC13611171; doi:10.3390/sports14090382)
Supplement: Supplementary file 1 [file sports-14-00382-s001.zip › sports-4451996-supplementary.pdf]

Variable	PC1 loading
Serve	−0.299
Volley	−0.309
Technique	−0.332
Accuracy	−0.288
Grip strength	−0.298
Standing long jump	−0.331
Reaction performance	−0.318
Throwing speed	−0.341
15-m sprint	0.353
Multiskill test	0.285

Table S1. Principal component loadings for the retained first principal component

Note. Principal component analysis was performed on the ten standardized performance variables (N = 113). Parallel analysis supported retention of one principal component. The first component had an eigenvalue of 3.620 and accounted for 36.2% of the total variance. The sign of principal component loadings is arbitrary; the opposite signs of the 15-m sprint and multiskill test reflect that lower completion times indicate better performance.
